# A retrospective, real‐world experience of perampanel monotherapy in patient with first new onset focal seizure: A Thailand experience

**DOI:** 10.1002/epi4.12555

**Published:** 2021-11-10

**Authors:** Yotin Chinvarun

**Affiliations:** ^1^ Department of Neurology Phramongkutklao Royal Army Hospital and Medical College Bangkok Thailand

**Keywords:** + antiepileptic drug, + Monotherapy, + New focal onset seizure, + Perampanel

## Abstract

**Objective:**

Real‐world data on efficacy and tolerability of perampanel (PER) monotherapy in treatment‐naïve patients with focal onset seizures (FOS) and/or focal‐to‐bilateral tonic‐clonic seizures (FBTCS) to assess efficacy effectiveness and tolerability.

**Methods:**

This is a retrospective review of study patients with new FOS with or without FBTCS, aged ≥15 years, who had been prescribed PER as monotherapy. Treatment outcome included retention rate, responder, and seizure‐free rate at observational point 3, 6, and 12 months (OP3, OP6, and OP12). Treatment‐emergent adverse events (TEAEs) and adverse drug reactions were recorded.

**Results:**

A total of 41 patients enrolled in the study (male:female; 17:22, mean age =46.1 ± 21.8 years), with new FOS and/or FBTCS. The proportions of individuals remaining on PER monotherapy at 3, 6, and 12 months were evaluated. The median PER dosage was 4 mg (range 2‐8 mg). The retention rates at OP3, OP6, and OP12 were 88%, 73%, and 61%, respectively. The seizure freedom rates at OP3, OP6, and OP12 were 78%, 80%, and 76%, respectively. About 14% had discontinued the PER monotherapy because of lack of efficacy. Sixteen individuals (41%) had TEAEs; common AEs were dizziness, somnolence, and ataxia; and only one case had depression. The AEs with somnolence and ataxia were found higher in elderly (15% and 30%) than adult patients (7% and 3%), respectively. Only 14% had intolerant adverse events, and it was found higher in elderly (23%).

**Significance:**

Real‐world data of PER monotherapy in treatment‐naïve patients with focal onset seizures demonstrated good effectiveness and a good safety profile at relatively low doses. By starting with low dosage and slow titration of PER help to minimize the impact of adverse effects, maximize adherence, and increase patient retention. PER has a once‐daily dosing schedule that supports patient adherence contributes to achieving seizure freedom.

**Key Points**

• PER monotherapy is an effective treatment in adult and elderly patient with first new onset FOS and/or FBTCS in routine clinical practice at relatively low doses with median PER daily dosage 4 mg (range 2‐8 mg).
• The retention rates at OP3, OP6, and OP12 were 88%, 73%, and 61%, respectively, and the seizure freedom rate in adult at OP3, OP6, and OP12 were 78%, 80%, and 76%, respectively, whereas the percentage of seizure‐free in elderly was seen at OP3, OP6, and OP12 was 85%, 91%, and 80%, respectively.
• With a low starting dose and utilizing a slow titration strategy is recommended to minimize the impact of adverse effects, maximize adherence, and increase patient retention
• PER monotherapy has a good safety profile and well tolerated, with the most common ADRs observed being, dizziness, ataxia, and somnolence.
• The long half‐life allows for once‐daily dosing that could also benefit patients who miss a treatment dose that promotes adherence contributes to achieving seizure freedom.

## 1 INTRODUCTION

Monotherapy may be preferable in some clinical practice settings because it reduced likelihood of adverse events, decreased risk of drug‐drug interactions, better compliance, easy to evaluate individual drugs, and low cost compared with polytherapy.^1^ The majority of patients with epilepsy respond to treatment with monotherapy: 47% of patients become seizure‐free with the first antiseizure drugs (ASMs) tried, and another 13% achieve freedom from seizures with the second monotherapy trial.^2^ With each subsequent ASM regimen trialed, the probability of achieving seizure freedom diminishes substantially; most patients who gain seizure control do so with the first or second ASM prescribed.^3^ Therefore, early selection of an effective ASM for initial monotherapy or as an early adjunctive therapy is critical for realizing the best possible therapeutic outcomes. To achieve a successful monotherapy in new onset epilepsy management includes the following: (1) select an efficacious ASMs for the specific seizure type; (2) choose an ASMs with a tolerable adverse effect and less toxicity profile; (3) easy to use ASMs such as once‐daily dosage to get a better compliance; and (4) titrate the ASMs slowly to the desired dose.

Perampanel is a first‐in‐class AMPA receptor antagonist approved for the treatment of epilepsy and has broad‐spectrum efficacy.^4^, ^5^, ^6^, ^7^ PER is indicated by the Food and Drug Administration (FDA) for treatment of focal onset seizures (FOS), with or without focal‐to‐bilateral tonic‐clonic seizures (FBTCS) in patients ≥4 years of age (monotherapy and adjunctive therapy) and adjunctive therapy in the treatment of primary generalized tonic‐clonic seizures in patients with epilepsy ≥12 years of age.^8^ In addition, PER has a once‐daily dosing schedule that supports patient adherence.^9^, ^10^, ^11^ PER was approved for monotherapy use for focal seizures in the United States.^12^ PER monotherapy has shown antiseizure effects in several animal models of epilepsy and status epilepticus,^13^ but there are limited data regarding clinical experience with PER when used as monotherapy in human. Real‐world evidence may be a useful approach to explore the feasibility of ASM monotherapy in the clinic. We report the results of a retrospective study evaluating PER monotherapy in the Thai patients with the first new focal onset seizure.

## 2 METHODS

### 2.1 Study design and population

This was a real‐world retrospective study at the Phramongkutklao hospital (PMK hospital) to investigate the dosage, efficacy, and safety of PER given as monotherapy in routine clinical care to patients with first new onset focal seizure. The data were collected retrospectively for individuals who presenting with the first new onset focal seizure who received PER as the first antiseizure medication (ASM) with monotherapy, when they have the 1^st^ seizure in between July 2015 and March 2020 at the comprehensive epilepsy center, Phramongkutklao hospital. By using PER as the 1^st^ ASM in patient presenting with the 1^st^ seizure with focal onset seizure, all had clinical information of the 1^st^ seizure with focal onset confirmed by EEG or 24 hours video‐EEG monitoring and mostly had MRI brain done, four cases had CT brain done, and only one case had no neuroimaging. Patients who presenting the first new onset seizure with focal onset were identified from electronic/paper medical and pharmacy records of individuals who attending at epilepsy clinic and were prescribed PER as the 1^st^ ASM monotherapy. Anonymized information was collected from medical records. Where applicable, independent Ethics Committee and regulatory authority review and approval were obtained in accordance with local legislation.

The patients were initiated on once‐daily oral PER 2 mg/d before bedtime for 2 weeks, and if there were no tolerability issues to occur, then the PER will be uptitrated to 4 mg/d as the minimal dosage. However, if the patient had in‐tolerability adverse effect, the patient was encouraged to taper down and to the previous tolerated dosage for another 2‐4 weeks and then the dosage will be titrated up again to 4 mg/d. The patients who tolerated PER at 4 mg/d, if they had any experienced seizures, then the PER will be gradually uptitrated to 6, 8, 10 mg and maximally to 12 mg/d, respectively, in every 2‐4 weeks. However, if the patient had intolerant adverse effect or the seizure worsen, then the PER will be taken off and changed to use other ASMs. The patients who had partial response to the PER, if the patients still were not achieved seizure‐free after trying the PER monotherapy, then the patient will be changed to use polytherapy or alternative monotherapy.

### 2.2 Data collection

Data for evaluation of clinical history, diagnosis, assessment of the therapeutic response from medical records, data on seizure frequency, and safety were collected. The EEG data and neuroimaging also were obtained.

### 2.3 Objectives and analyses

All individuals with first new focal seizure who had received PER monotherapy were included and had seizure frequency data available were included in the full analysis set.

The primary objective of the study was to assess the retention rate of PER when given as monotherapy in routine clinical care. The proportions of individuals remaining on PER monotherapy (retention rates) at 3, 6, 12 months were evaluated as primary endpoints. The patients needed to be on PER monotherapy for at least three months have to be analyzed. The denominators for these retention rates were the numbers of individuals who could have been exposed for each period of time.

The following secondary endpoints, relating to changes in seizure frequency, were assessed in the full analysis set: the proportion of individuals who were seizure‐free for at least 3, 6, and 12 months while receiving PER monotherapy and changed in seizure frequency between pre‐PER baseline. Seizure freedom was defined as complete seizure control on PER monotherapy since the prior visit, which for the 12 months visit meant no seizures during at least the prior 6 months, and for the 3‐ and 6 months visits meant no seizures since baseline or 3 months visit, respectively. The changes in seizure frequency were assessed as the following: median percent change in seizure frequency per 30 days, proportions of individuals with a reduction in seizure frequency of 50% (50% responder rate), reduction in seizure frequency of 75% (75% responder rate), and proportions of individuals with no changed or a worsening of seizure frequency. The maximum and the median doses of PER were recorded.

### 2.4 Safety assessments

The treatment‐emergent adverse events (TEAEs) and serious TEAEs, assessed in the safety set, were determined by the type and frequency of all TEAEs and discontinuations related to PER that had been recorded from the initiation of PER monotherapy until the last follow‐up after the last dose of PER monotherapy.

## 3 RESULTS

### 3.1 Study population and baseline characteristics

**FIGURE 1:**
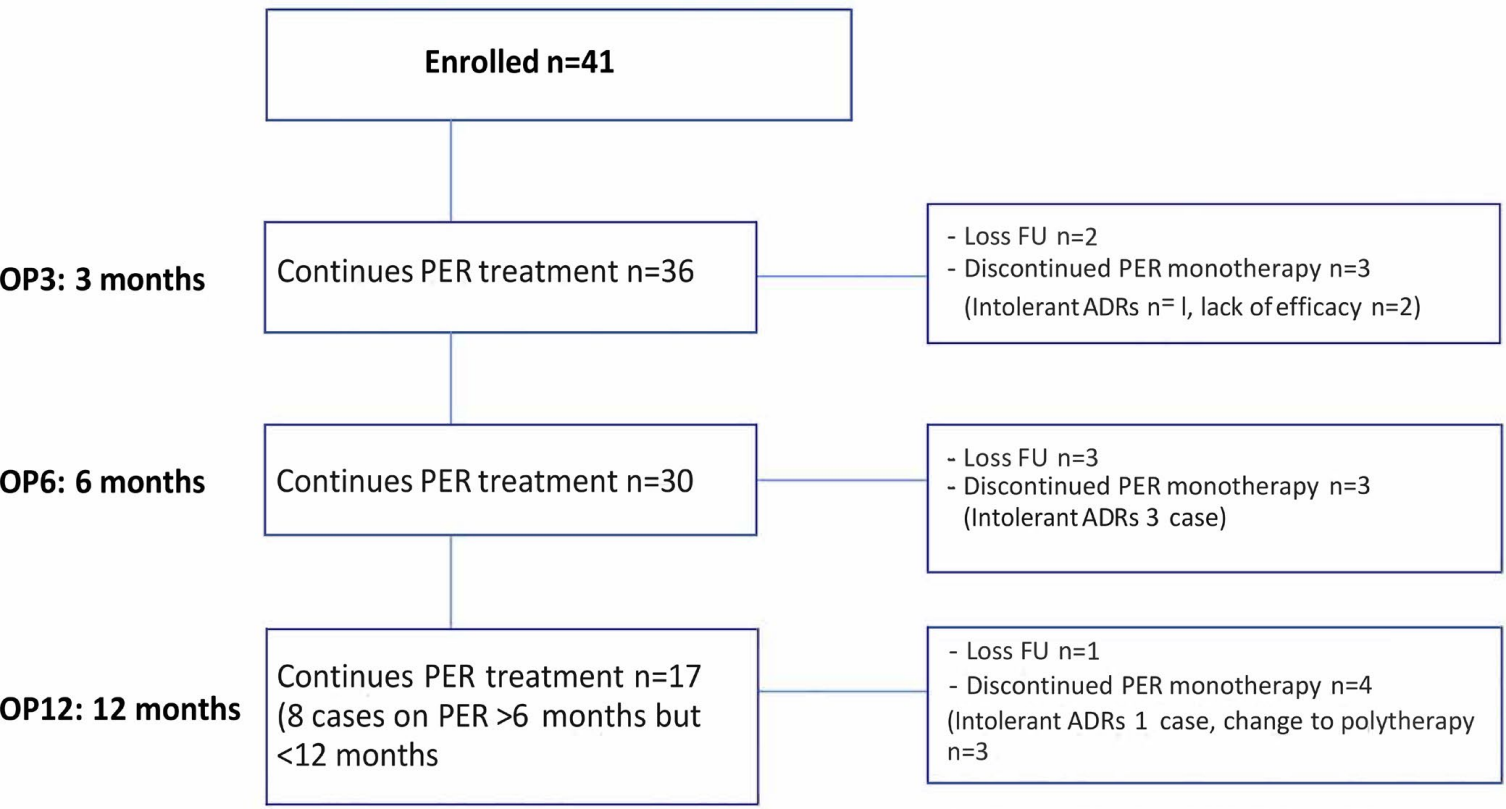
Illustration of the number of patients (n = 41 cases) evaluated at each visit who have been treated with perampanel (PER) monotherapy at some point during the first 12 mo

A total of 41 patients (male:female; 17:24 cases) from PMK hospital were enrolled in the study. The mean age at the start of PER monotherapy was 46 years (range 15‐88 years). All had clinical diagnosis of new focal onset seizure confirmed with clinical seizure, EEG/video‐EEG monitoring (VEM), and most of the cases had 24 hours and MRI brain study done (only four had CT brain and one case had no neuroimaging). Patients had a median epilepsy duration of 107 days (range =1 day‐5 years) (Table 1). Thirty‐six patients were maintained on PER monotherapy for the first 3 months, thirty cases maintained on PER for 6 months, and seventeen cases on PER for 1 years (whereas 8 cases were on PER more than 6 months, but less than a year) (Figure 1). The median PER dosage was 4 mg (range 2‐8 mg). The 4 mg was the most common dose (61%), followed by 2 mg (20%), 6 mg (17%), and 8 mg (2%). In elderly patients (>60 years), the daily dose of PER was similar as adult (median dose of 4 mg in patients aged >60 years vs 4 mg in younger patients). Titration was considered fast (2 mg every 2 weeks or less) in 37 patients (90%) and slow (>2 weeks) in 4 patients (10%).

**TABLE 1:** Demographic and clinical characteristics (n = 41)

	Perampanel monotherapy for new onset focal seizure
Age, mean (range) years	46.1 ± 21.8 (15‐88)
<60 y, n (%)	28 (68%)
>60 y, n (%)	13 (32%)
Gender n (%)	
Male	17 (41%)
Female	24 (59%)
Seizure onset, y (min, max)	1 d–5 y
Seizure frequency per month, median (min, max)	1‐3/mo, 1.7 (1‐14)
Focal seizures, n (%)	
Focal onset with awareness	‐
Focal onset with impaired awareness	30 (73%)
Evolving to bilateral tonic‐clonic seizure	15 (37%)
History of seizure clusters and/or status epilepticus	No
Etiology not known	19 (46%)
Etiology known	
Cerebrovascular	3 (7%)
Neurodegenerative	2 (5%)
Cranial trauma	1 (2%)
Cerebral neoplasm	2 (5%)
Malformations of cortical development (MCD)	4 (10%)
Mesial temporal sclerosis	2 (5%)
Hippocampal atrophy	6 (15%)
AVM	1 (2%)
Other	1 (2%)

### 3.2 Retention rates

**FIGURE 2:**
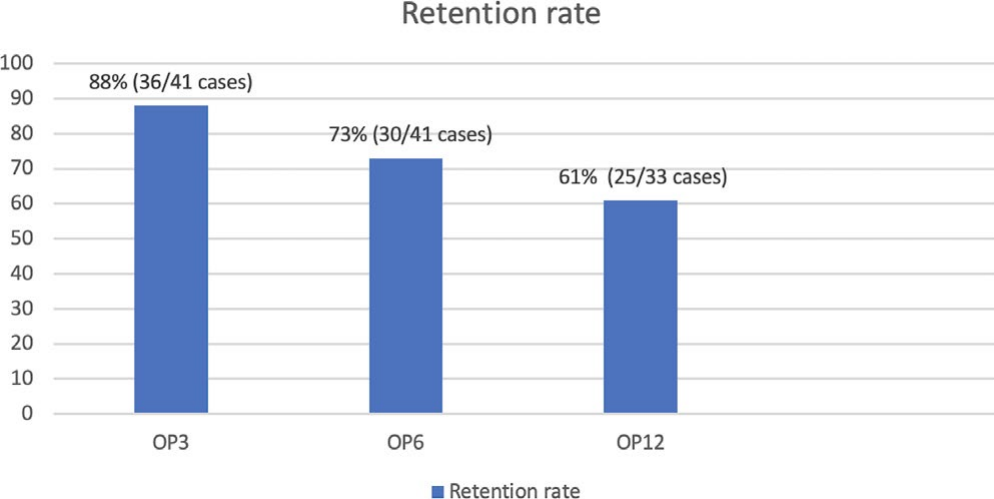
Retention rates on perampanel monotherapy

The median length of exposure to PER monotherapy was 8 months (range =3‐12 months). At OP3, the retention rates were 88.0% (two cases lost follow‐up, discontinued PER monotherapy n = 3; one case had intolerant ADRs and two cases had lack of efficacy). At OP6, the retention rates were 73% (three cases lost follow‐up, discontinued PER monotherapy n = 3; all had intolerant ADRs). The retention rates at OP12 were 61.0%, and only 17 cases were available for analysis (8 cases on PER more than 6 months, but less than 12 months). One case lost follow‐up, and four cases were discontinued from PER monotherapy (one case had intolerant ADRs, and 3 cases change to duotherapy for better seizure control), (Figure 2).

### 3.3 Changes in seizure frequency

#### 3.3.1 Changes in seizure frequency at different observational point

**FIGURE 3:**
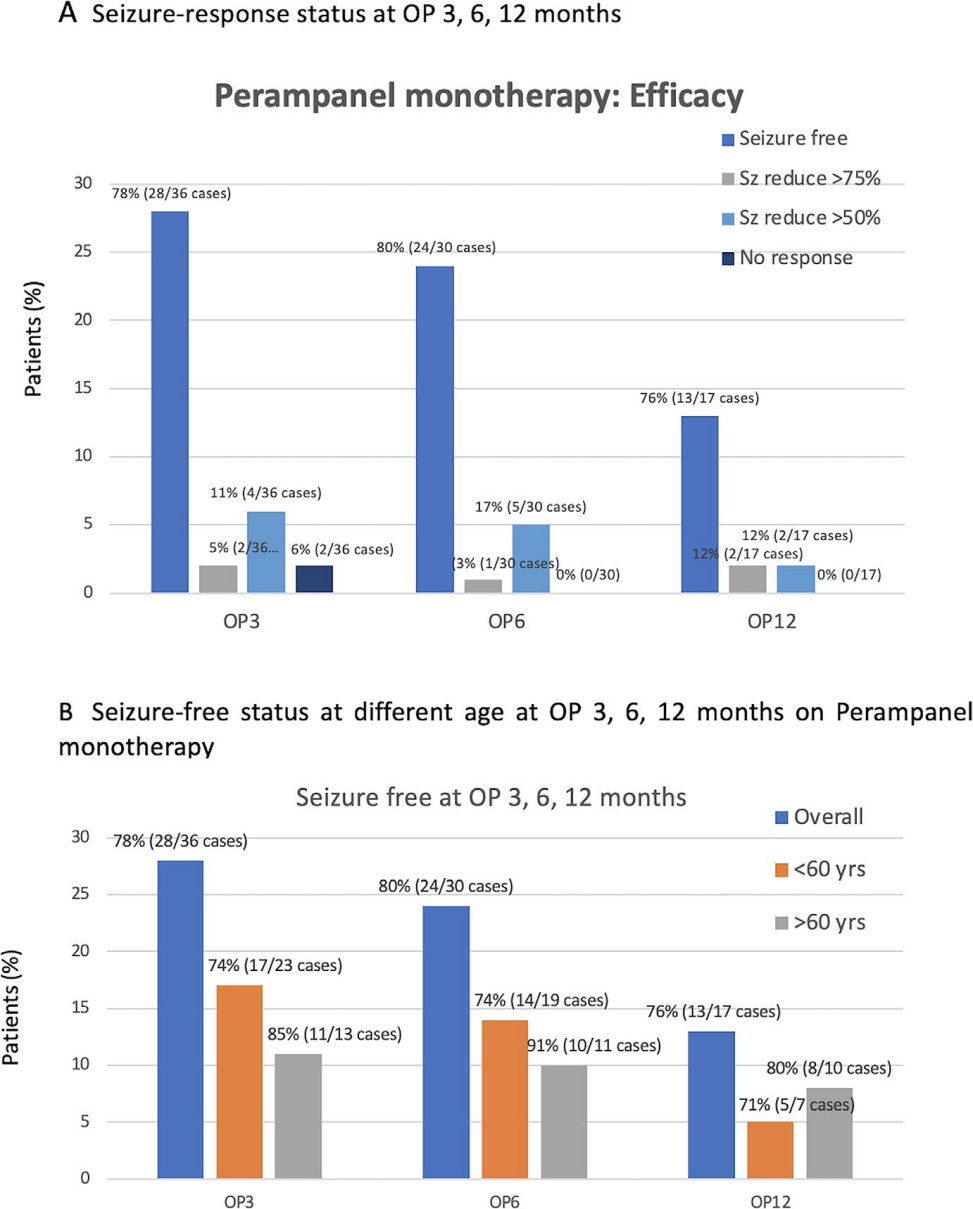
Seizure‐response status and seizure‐free status at different age at OP3, 6, 12 mo on perampanel monotherapy

**FIGURE 4:**
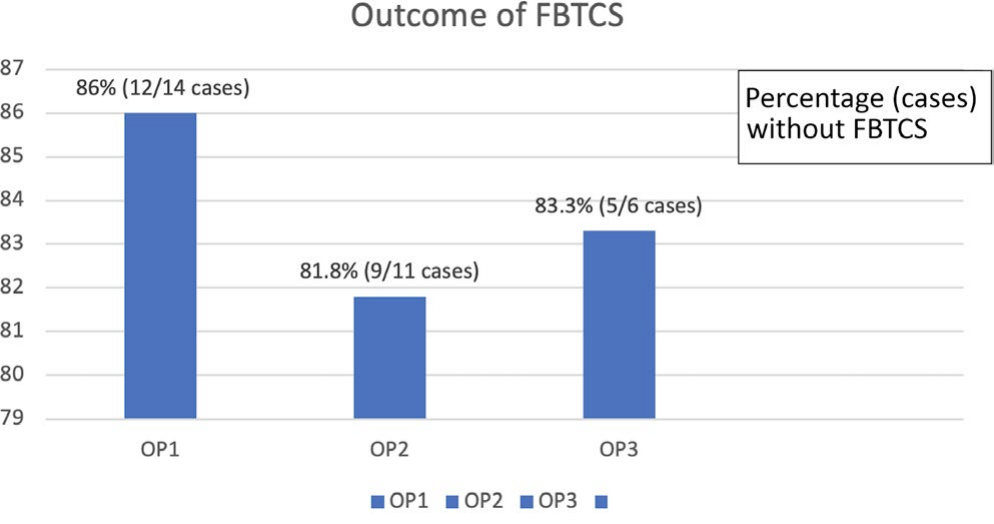
Outcome of FBTCS

Of the 41 individuals who had seizure frequency data available and were thus included in the full analysis set, 78% (n = 28/36 cases) were seizure‐free at OP3 while receiving PER monotherapy. The median percent reductions in seizure frequency 75% and 50% responder rates were 5% and 11%, respectively, whereas 6% was nonresponsive. At observation point at 6 months (OP6), 80% (24/30 cases) were seizure‐free at observation point at 6 months (OP6), and the patients with median percent reductions in seizure frequency 75% and 50% were found as 3% and 17%, respectively. At the OP12, 76% (13/17 cases) were seizure‐free and patients with median percent reductions in seizure frequencies 75% and 50% were equal around 12% (Figure 3A). Regarding the 14 patients with focal‐to‐bilateral tonic‐clonic seizures (FBTCS), 86.0% (12/14 cases) were seizure‐free at 3 months, 81.8% (9/11 cases) at 6 months, and 83.3% (5/6 cases) at 12 months (Figure 4).

#### 3.3.2 Changes in seizure frequency in elderly

The percentage of changing in seizure frequency was seen differently in the elderly (Figure 3B), and the percentage of seizure‐free in elderly (age >60 years old) was higher than the patient who were ≦60 years old. The percentage of seizure‐free in elderly seen at OP3, OP6, and OP12 was 85%, 91%, and 80%, respectively, whereas the percentage of seizure‐free in adult seen at OP3, OP6, and OP12 was 78%, 80%, and 76%, respectively.

### 3.4 Safety and tolerability

Sixteen individuals (41%) had treatment‐emergent adverse events (TEAEs) during monotherapy PER treatment (Table 2). The most common TEAEs were dizziness, somnolence, ataxia, and only one case had depression. The TEAEs with somnolence and ataxia were found more common in the elderly (patients >60 years old) 15% and 30% than in adult patients (≦60 years old) 7% and 3%, respectively. Only 14% (5 cases) had intolerant adverse events, which is more common in the elderly. There was no any serious TEAE occurred in both groups during PER monotherapy.

**TABLE 2:** Incidence of treatment‐emergent adverse event (TEAEs)

	Overall (n = 39), n (%)	Age <60 y (n = 26), n(%)	Age >60 y (n = 13), n(%)
Any AEs	16 (41)	10 (38)	6 (46)
Serious AEs	0	0	0
Severe AEs	0	0	0
Death	0	0	0
Discontinuation due to AEs	6 (15)	3 (12)	3 (23)
Incidence of individual AEs			
Dizziness	11 (27)	9 (32)	2 (15)
Somnolence	4 (10)	2 (7)	2 (15)
Ataxia	6 (15)	2 (7)	4 (30)
Dry mouth	1 (2)	1 (4)	0
Depression	1 (2)	1 (4)	0
Confusion	1 (2)	1(4)	0

Abbreviation: AEs, Adverse effects; TEAE, Treatment‐emergent adverse event.

## 4 DISCUSSION

This real‐world retrospective study included 41 individuals with new focal onset epilepsy who received PER monotherapy as the first ASM of routine clinical care. The patients in this study represent the real‐world epilepsy heterogeneous population with new FOS and/or FBTCS in routine clinical practice, in which the seizure onset range from 1 day‐5 years. Because antiseizure drug polytherapy is often associated with increased toxicity, nonadherence, drug interaction, and cost,^14^ monotherapy may be preferable in some clinical practice settings and might be helping to improve compliance due to PER once‐daily dosing.

There is a limited information regarding clinical experience with PER monotherapy, recent studies are encouraging in suggesting that PER might be useful as a monotherapy in a selected group of patients. A retrospective study^15^ evaluated the efficacy of PER monotherapy of 60 patients. Retentions rates of PER treatment at 3 and 6 months were 95% and 74%, respectively. There were 40 patients included in the full analysis set, more than half (n = 22; 55%) were seizure‐free for at least 3 months at any time while receiving PER as a primary or secondary monotherapy. At the study cutoff date, there were 41 patients (68%) continuing PER monotherapy. Nineteen patients (32%) had discontinued the PER monotherapy, most commonly due to lack of efficacy (n = 11) or AEs (n = 6). In our study, the retention rates are similar after approximately 3, 6, 12 months (OP3, OP6, OP12) of PER monotherapy, and the retention rates were 88%, 73%, and 61%, respectively. About 14% had discontinued the PER monotherapy because of lack of efficacy and had been changed to polytherapy or alterative monotherapy. Five cases (14%) had an intolerant ADR.

From the FREEDOM study,^16^ a Phase III, open‐label study in Japan and South Korea of the efficacy and safety of PER monotherapy in patients with FOS with or without FBTCS for 26 weeks (N = 73). All patients were newly diagnosed with epilepsy or had experienced seizure recurrence after a period of remission at least 2 years after the cessation of the last ASM treatment. Patients were treated with 4 mg/day PER, which could be titrated to 8 mg/day following a seizure. PER monotherapy was found to be efficacious, with a 63.0% seizure freedom rate achieved in patients who were maintained on the 4 mg/day dose and 74.0% overall and seizure freedom rate at 6 months was 80% and at 6 months was 76%. Compared with the previous study, the responder rates in our study in a newly diagnosed focal onset epilepsy for all seizures were similar as high as 78% at 3 months, 80% at 6 months, and 76% at 12 months. The seizure‐free rate in our study was sustained within the period of 12 months. The most common PER dosage in our study was 4 mg (61%), followed by 2 mg (20%), 6 mg (17%), and 8 mg (2%). In elderly patients (>60 years), the daily dose of PER was similar as adult (median dose of 4 mg in patients aged >60 years vs 4 mg in younger patients). This study showed that the optimal maintenance dose for PER for most patients is either 4 mg or 6 mg, although there were a few cases still get beneficial when on PER monotherapy at 2 mg daily dosage. This dosage offers effective seizure reduction while minimizing adverse events in most patients. Titration was considered fast (2 mg every 2 weeks or less) in 37 patients (90%) and slow (>2 weeks) in 4 patients (10%). Our study found that a slow titration strategy for PER might be needed in some patients such as in elderly, by increasing the daily dose by 2 mg every 4 weeks or at even longer intervals. The consideration of a lower starting dose (1 mg/day) for elderly, with slow uptitration of PER dose at 2‐ to 4 weeks intervals might be needed to explore. Where PER suspension/granule formulations are available, an alternative strategy is to increase the PER dose by 1 mg every 2 weeks might help to improve tolerability. Using strategy with a low starting dose and utilizing a slow titration could be helping to minimize the impact of adverse effects, maximize adherence, and increase patient retention. PER requires once‐daily dosing, which has a long half‐life, has been demonstrated to be an effective strategy for improving patient adherence, and may be beneficial if a patient misses a dose.

The ASM options are limited in elderly patients because of safety concerns, but our study showed that PER in the new onset focal seizure has a favorable efficacy and safety profile in the elderly. However, a lower starting dose (≤1 mg/day) and a slow titration might need to consider for elderly.

PER demonstrated efficacy against the focal‐to‐bilateral tonic‐clonic (FBTCS) seizures.^17^ In our study, analysis of the pooled data at different observational point suggested that PER had a high efficacy against secondarily generalized tonic‐clonic seizures and was sustainable up to a year.

Overall, PER monotherapy was generally well tolerated, with most reported adverse events being mild in nature. Most common ADRs leading to discontinuation in focal onset seizure studies were dizziness, somnolence, vertigo, aggression, anger, ataxia, blurred vision, irritability, and dysarthria. The most common adverse reactions leading to discontinuation in the generalized onset tonic‐clonic seizure study were vomiting and dizziness.^18^, ^19^ In our study, PER monotherapy was generally well tolerated with a frequency of mild ADRs (41% in the overall population). At doses of 4‐8 mg/day, treatment was well tolerated, and not any concern of safety signals was identified in our study. The withdrawal rate due to TEAEs was very low, only 14% withdrawn because of intolerant adverse events. Most common TEAEs were dizziness, somnolence, and ataxia; only one case had depression. A study found that the dose dependency was observed in the occurrence of serious and nonserious psychiatric or behavioral adverse reactions in pooled data of Phase III focal onset seizure studies. The inappropriate behavioral or psychiatric reactions were seen in 5.2% (n = 9) of patients in 4 mg/d, 12.3% (n = 53) in 8mg/d, and 20.4% (n = 52) in 12 mg dose versus 5.7% (n = 25) [43]. The most common reported psychiatric or behavioral reactions were irritability, aggression, skin laceration, anger, agitation, and abnormal behavior. Our study had only a few psychiatric ADRs because this is a real‐world practice; therefore, the patients who were vulnerable to have psychiatric problems were not enrolled in the study. The somnolence and ataxia most commonly documented ADR in the elderly population (patients >60 years old than the patients ≦60 years old). However, the incidence of dizziness was more commonly found in the adult group. The elderly had intolerant adverse events much higher than the adult group (23% vs 14%). Reiterating the importance of taking PER immediately before going to bed was recommended when somnolence or dizziness occurs. Also, if the adverse effect develops during the maintenance period, reduce PER dose for a short period of time until the adverse effect resolves, the dose may be uptitrated again slowly in every 2‐4 weeks once the patient is better tolerating the medication.

## 5 IN SUMMARY

This study provided an insight into the feasibility of PER monotherapy in a new focal onset epilepsy in real‐world settling. PER is an effective treatment when used as monotherapy at relatively low doses with median PER daily dosage 4 mg (range 2‐8 mg) in adult and elderly patients with FOS and/or FBTCS in routine clinical practice. The high retention rate was found in this study, which reflects both its tolerability and its effectiveness, combined with its broad‐spectrum mechanism of action supports that patients with focal onset epilepsy in adult and elderly get the benefit from PER monotherapy. With a low starting dose and utilizing a slow titration strategy is recommended to minimize the impact of adverse effects, maximize adherence, and increase patient retention. Clinical trials show that PER is well tolerated, with the most common ADRs observed being, dizziness, ataxia, and somnolence. The long half‐life allows for once‐daily dosing that could also benefit patients who miss a treatment dose that promotes adherence contributes to achieving seizure freedom.

## 6 LIMITATIONS

This real‐world study has potential limitations, retrospective design, and small population size and did not involve a comparator arm.

## CONFLICT OF INTEREST

Yotin Chinvarun has received speaker's honoraria from Eisai Pharmaceutical and had participated in advisory boards for Eisai. I confirm that I have read the Journal's position on issues involved in ethical publication and affirm that this report is consistent with those guidelines.

## AUTHOR CONTRIBUTION

Yotin Chinvarun contributed to the study design, protocol development, data interpretation and analysis, preparation of the manuscript, and submit this article for publication.
